# Associations of hypoglycemia, glycemic variability and risk of cardiac arrhythmias in insulin-treated patients with type 2 diabetes: a prospective, observational study

**DOI:** 10.1186/s12933-021-01425-0

**Published:** 2021-12-24

**Authors:** Andreas Andersen, Jonatan I. Bagger, Samuel K. Sørensen, Maria P. A. Baldassarre, Ulrik Pedersen-Bjergaard, Julie L. Forman, Gunnar Gislason, Tommi B. Lindhardt, Filip K. Knop, Tina Vilsbøll

**Affiliations:** 1 Clinical Research, Steno Diabetes Center Copenhagen, University of Copenhagen, Borgmester Ib Juuls Vej 83, 2730 Herlev, Denmark; 2 Center for Clinical Metabolic Research, Herlev and Gentofte Hospital, University of Copenhagen, Hellerup, Denmark; 3 Department of Cardiology, Herlev and Gentofte Hospital, University of Copenhagen, Hellerup, Denmark; 4 Department of Medicine and Aging Sciences, G. d’Annunzio University, Chieti, Italy; 5 Department of Clinical Medicine, Faculty of Health and Medical Sciences, University of Copenhagen, Copenhagen, Denmark; 6 Department of Endocrinology and Nephrology, Nordsjællands Hospital Hillerød, University of Copenhagen, Hillerød, Denmark; 7 Deparment of Biomedical Sciences, Faculty of Health and Medical Sciences, University of Copenhagen, Copenhagen, Denmark; 8 The Danish Heart Foundation, Copenhagen, Denmark; 9 Novo Nordisk Foundation Center for Basic Metabolic Research, Faculty of Health and Medical Sciences, University of Copenhagen, Copenhagen, Denmark

**Keywords:** Type 2 diabetes, Insulin treatment, Hypoglycemia, Glycemic variability, Cardiac arrhythmias

## Abstract

**Background:**

Insulin-treated patients with type 2 diabetes (T2D) are at risk of hypoglycemia, which is associated with an increased risk of cardiovascular disease and mortality. Using a long-term monitoring approach, we investigated the association between episodes of hypoglycemia, glycemic variability and cardiac arrhythmias in a real-life setting.

**Methods:**

Insulin-treated patients with T2D (N = 21, [mean ± SD] age 66.8 ± 9.6 years, BMI 30.1 ± 4.5 kg/m^2^, HbA1c 6.8 ± 0.4% [51.0 ± 4.8 mmol/mol]) were included for a one-year observational study. Patients were monitored with continuous glucose monitoring ([mean ± SD] 118 ± 6 days) and an implantable cardiac monitor (ICM) during the study period.

**Results:**

Time spend in hypoglycemia was higher during nighttime than during daytime ([median and interquartile range] 0.7% [0.7–2.7] vs. 0.4% [0.2–0.8]). The ICMs detected 724 episodes of potentially clinically significant arrhythmias in 12 (57%) participants, with atrial fibrillation and pauses accounting for 99% of the episodes. No association between hypoglycemia and cardiac arrhythmia was found during daytime. During nighttime, subject-specific hourly incidence of cardiac arrhythmias tended to increase with the occurrence of hypoglycemia (incident rate ratio [IRR] 1.70 [95% CI 0.36–8.01]) but only slightly with increasing time in hypoglycemia (IRR 1.04 [95% CI 0.89–1.22] per 5 min). Subject-specific incidence of cardiac arrhythmias during nighttime increased with increasing glycemic variability as estimated by coefficient of variation whereas it decreased during daytime (IRR 1.33 [95% CI 1.05–1.67] and IRR 0.77 [95% CI 0.59–0.99] per 5% absolute increase, respectively).

**Conclusions:**

Cardiac arrhythmias were common in insulin-treated patients with T2D and were associated with glycemic variability, whereas arrhythmias were not strongly associated with hypoglycemia.

*Trial registration*: NCT03150030, ClinicalTrials.gov, registered May 11, 2017. https://clinicaltrials.gov/ct2/show/NCT03150030

**Supplementary Information:**

The online version contains supplementary material available at 10.1186/s12933-021-01425-0.

## Background

Patients with type 2 diabetes have an excess cardiovascular and all-cause mortality compared to patients without diabetes [1, 2]. Epidemiological data suggest that this excess mortality can be reduced by lowering HbA_1c_, however, randomized controlled trials have failed to demonstrate a beneficial effect of strict glycemic control [3]. It has been suggested that the potential cardiovascular benefit from near-normal HbA_1c_ may be outweighed by a negative effect of an increased risk of hypoglycemia, especially in older patients with long diabetes duration and high cardiovascular risk [4]. Consequently, current guidelines for treatment of type 2 diabetes emphasize that near-normal HbA_1c_ should only be targeted when it can be achieved safely without any substantial increase in episodes of hypoglycemia [4].

The deleterious effect of hypoglycemia on the cardiovascular system is thought to be mediated through multiple pathways, including blood coagulation abnormalities, inflammation, endothelial dysfunction and abnormal cardiac rhythm [5]. Increased risk of cardiac arrhythmias during hypoglycemia has been investigated since an association was first reported in 1991 [6]. Studies of experimentally induced hypoglycemia almost consistently link hypoglycemia to abnormal cardiac repolarization constituting a strong marker of risk of ventricular arrhythmias [7]. However, real-life, prospective, observational studies with four to five days of continuous glucose monitoring (CGM) and concomitant Holter monitoring have only provided sparse evidence for an association between hypoglycemia and clinically relevant arrhythmias [8–11]. Hence, studies in high-risk individuals with type 2 diabetes applying long-term monitoring are necessary to improve our understanding of the relation between hypoglycemia and cardiac arrhythmias [12].

During the last decades, the options for clinical long-term monitoring of glycemia and cardiac rhythm have continuously evolved [13, 14]. CGM devices have improved their accuracy and have become more comfortable for patients to use [15]. Furthermore, the development of implantable cardiac monitors (ICM) allows long-term cardiac monitoring in a real-life setting, enabling the detection of clinically important but infrequent events [16].

In the present study, we investigated the prevalence of clinically significant arrhythmias in insulin-treated patients with type 2 diabetes and the association between arrhythmic events and episodes of hypoglycemia and glycemic variability. This was achieved by employing CGM and ICM concomitantly for long-term glycemic and cardiac monitoring in a real-life setting.

## Methods

### Approvals and registrations

The study was carried out at Steno Diabetes Center Copenhagen and Center for Clinical Metabolic Research, Gentofte Hospital, University of Copenhagen, Hellerup, Denmark. The study was approved by the Scientific Ethical Committee of the Capital Region of Denmark (ID No. H-16046212) and the Danish Data Protection Agency (ID No. HGH-2017-030) and registered at ClinicalTrials.gov (NCT03150030). The study was conducted in accordance with the Declaration of Helsinki and oral and written consent was obtained from all participants prior to inclusion in the study.

### Design and study population

The study was a one-year, prospective, observational study including patients with insulin-treated type 2 diabetes with HbA_1c_ ≤ 7.5% (58 mmol/mol) and a least one microvascular or macrovascular complication. Information regarding complications were obtained from medical records, blood and urine samples as well as interview of the patients at the screening visit. Microvascular complications were defined as: (1) nephropathy (creatinine > 130 μmol/L and/or albuminuria), (2) retinopathy (moderate or severe), and (3) neuropathy (biothesiometry > 25 mV). Macrovascular complications were defined as: (1) coronary artery disease (previous acute myocardial infarction, unstable angina and stable angina), (2) cerebrovascular disease (previous stroke or transient ischemic attack), and (3) peripheral artery disease (previous intermittent claudication or prior acute ischemia). Patients were recruited from the diabetes outpatient clinics at Steno Diabetes Center Copenhagen, and Nordsjællands Hospital Hillerød, Denmark. All recruited patients continued their regular visits in the diabetes outpatient clinic. Patients with previous cardiac arrhythmia, implantable cardiac defibrillator or pacemaker, severe heart failure (left ventricular ejection fraction < 25%, structural heart disease and thyroid dysfunction (except for well-regulated levothyroxine-substituted hypothyroidism) were excluded. Patient visits were planned at 0, 1, 3, 6, 9 and 12 months after initiation of CGM and ICM data capture and included measurement of blood pressure, electrocardiography, urine sample, blood samples and echocardiography (0 and 12 months only). Left ventricular ejection fraction was evaluated by Simpson’s biplane method. Peak early mitral inflow velocity was measured in four-chamber view using pulsed-wave Doppler with the sample volume placed between the tips of the mitral valve leaflets. Mitral annular peak early diastolic velocity was estimated by pulse-waved tissue Doppler with the sample volume placed in the septal and lateral mitral annulus in four-chamber view.

### Continuous glucose monitoring

For evaluation of glycemia, patients were monitored with a blinded CGM system (iPro2, Medtronic, Minneapolis, MN, USA). The system allows up to six days of monitoring per sensor and has been validated elsewhere [17]. Patients underwent four consecutive 6-days periods of monitoring during the first and last month of the study and one 6-day period of monitoring per month during the 10 months in between. Participants were educated in the CGM system and obtaining four self-measured blood glucose measurements per day with a Contour XT glucometer (Ascensia, Basel, Switzerland) for the purpose of calibration. All recordings were manually validated, and patients were instructed to correct episodes of hypoglycemia by ingestion of carbohydrates. In case of sensor malfunction, insufficient calibration or poor data quality, the monitoring period was repeated. All results on glycemia were obtained from the CGM recordings and data were reported in accordance with recent consensus on CGM data reporting [18]. Four or more consecutive measurements of plasma glucose < 3.9 mmol/L or < 3.0 mmol/L was considered a level 1 or level 2 hypoglycemic episode, respectively [18]. Severe hypoglycemia (level 3) was defined as needing assistance. Target range was defined as plasma glucose 3.9–10.0 mmol/L and hyperglycemia as plasma glucose > 10.0 mmol/L. The terms time in target range, time in hypoglycemia and time in hyperglycemia correspond to time in range (TIR), time below range (TBR) and time above range (TAR). The nomenclature varies between consensus reports [18, 19]. Glycemic variability was evaluated by coefficient of variation (CV) and standard deviation (SD) as recommended, and stable glucose levels were defined as CV < 36%. The two-hour absolute excursion in plasma glucose was estimated as the difference between the peak and nadir plasma glucose value within same and previous hour. Daytime was defined as 6 am to midnight and nighttime as midnight to 6 am [18].

### Cardiac monitoring

Three weeks prior to the initiation of the one-year observational period, an ICM (Reveal LINQ, Medtronic, Minneapolis, MN, USA) was placed subcutaneously during local anesthesia. Home monitor systems were set up for daily, automated transmissions. An algorithm for tachycardia detection rate was designed to maximize the sensitivity for tachyarrhythmias in balance with avoidance of false positive detections of sinus tachycardia using up the ICM memory (Additional file 1). Bradycardia detection was set to eight consecutive beats with heart rate of ≤ 30 beats per min. Pause detection was set to ≥ 3 s without any QRS complexes. The ICM identified atrial fibrillation ≥ 6 min by a pattern-recognition algorithm based on beat to beat variability [20]. Details on ICM setup is presented in the supplemental material. All events were manually evaluated by the first author (AA) and in case of any uncertainty, an experienced cardiac electrophysiologist (TBL) made the final decision. Clinically relevant arrhythmias were defined by study protocol as atrial fibrillation and atrial flutter, brady-arrhythmias and tachy-arrhythmias. If a serial of events with atrial fibrillation or atrial flutter was recorded, a new event was only registered after at least two hours without reports of atrial fibrillation or atrial flutter. Clinically relevant brady-arrhythmias were defined as sinus arrest for more than three seconds, frequency below 30 beats per min, or high-grade atrioventricular block including Mobitz Type II and third-degree atrioventricular block. Clinically relevant tachy-arrhythmias were defined as sustained ventricular tachycardia (duration > 30 s) and non-sustained ventricular tachycardia.

### Statistics

Statistical analyses were performed with SAS studio version 3.8 (SAS Institute Inc., Cary, NC, USA). Data that followed an approximate normal distribution were summarized as mean ± SD, while skewed data were summarized as median (interquartile range). We aimed at relating glycemic characteristics to events of cardiac arrhythmia in continuous time so that we could separate the effect of within-subject changes in glycemic predictors from the between-subjects effect of differences in glycemic characteristics which is prone to confounding. To this end, episodes of cardiac arrhythmia were summarized by the hour and per nighttime/daytime period and analyzed as a binary outcome (event(s) vs no events). Glycemic data were summarized as mean plasma glucose, time in hypoglycemia (minutes), time in hyperglycemia (minutes), occurrence of hypoglycemia (binary) and occurrence of hyperglycemia (binary) per hour for each participant, by delta plasma glucose calculated as the difference between the peak value and the nadir value over two hours as well as SD and CV per nighttime and daytime period. Daytime and nighttime were analyzed separately. To separate the between-subject effect from the within-subject effect of each predictor, we computed averages over the study period and hourly deviations from average for each subject [21]. Finally, the predictors were entered as fixed effects in a logistic regression-type generalized linear mixed model which further included subject ID as a random effect. Results are presented as subject-specific odds ratios for within-subject effects with 95% CI. Due to the overall low occurrence of arrhythmia these are interpreted as incidence rate ratios (IRR) with 95% CI. Considering the exploratory nature of this study, *P* values are not reported.

## Results

### Study participants

Twenty-one patients with insulin-treated type 2 diabetes (3–36 years of duration) were included in the study (Table 1). All patients had microvascular complications and four patients had macrovascular complications (Table 1). No patients were lost to follow up.

**Table 1:** Baseline characteristics (N = 21)

Age (years)	66.8 (9.6)
Female	6 (28.6%)
BMI (kg/m^2^)	30.1 (4.5)
Smoking	
Previous	10 (48%)
Current	3 (14%)
HbA1c (%)	6.8 (0.5)
HbA1c (mmol/mol)	51.0 (4.8)
FPG (mmol/L)	7.5 (1.8)
Insulin treatment	21 (100%)
Basal	11 (52%)
Basal/bolus	5 (24%)
Insulin mix	4 (19%)
Insulin mix/bolus	1 (5%)
Daily insulin dose (IU)	40 (25–58)
Oral glucose-lowering drugs	13 (62%)
Metformin	10 (48%)
SGLT2i	4 (19%)
DDP-4i	3 (14%)
Diabetes duration (years)	18.2 (7.9)
Impaired awareness	6 (28.6%)
Microvascular complications	
Neuropathy	18 (86%)
Retinopathy	7 (33%)
Nephropathy	9 (43%)
Macrovascular complications	
Coronary artery disease	2 (10%)
Cerebrovascular disease	1 (5%)
Peripheral artery disease	1 (5%)
Hypertension	18 (86%)
Beta blocker	4 (19%)
Non-dihydropyridine calcium channel blocker	1 (5%)
Systolic blood pressure (mmHg)	145 (133–148)
Diastolic blood pressure (mmHg)	80 (75–85)
Heart rate (bpm)	64.3 (10.6)
Creatinine (µmol/L)	87 (71–109)
Potassium (mmol/L)	4.2 (0.4)
Echocardiographic measures	
Left ventricular ejection fraction (%)	58 (7.2)
E/e′ (average of septal and lateral)	10.5 (2.7)

Binary data are presented as N (%) and continuous variables are presented as mean with SD in parentheses or median with interquartile range in parentheses

bpm, beats per min; DDP-4i, dipeptidyl peptidase-4 inhibitor; E, peak early mitral inflow velocity; e′, mitral annular peak early diastolic velocity; FPG, fasting plasma glucose; SGLT2i, sodium-glucose transport protein 2 inhibitor

### Continuous glucose monitoring

**Fig. 1:**
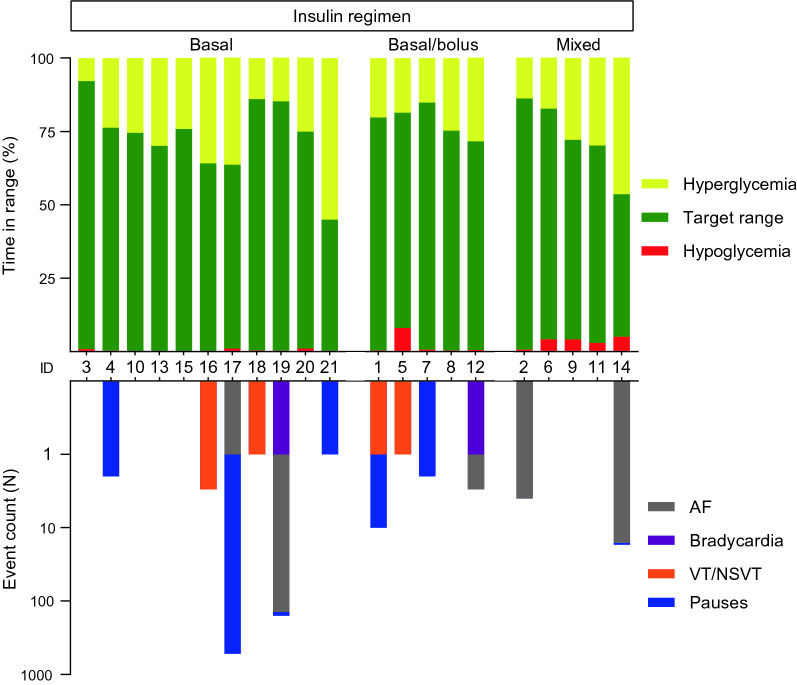
Individual time in range and arrhythmic event count. Individual time in range and arrhythmic event count for each participant displayed according to insulin regimen: (1) basal insulin only, (2) combination therapy with basal and bolus insulin, and (3) premixed basal and bolus insulin in a fixed ratio. Glycemic ranges defined as: (1) hypoglycemia (< 3.9 mmol/L), (2) target range (3.9–10.0 mmol/L), and (3) hyperglycemia (> 10.0 mmol/L). Event counts are displayed on an antilog scale

**Fig. 2:**
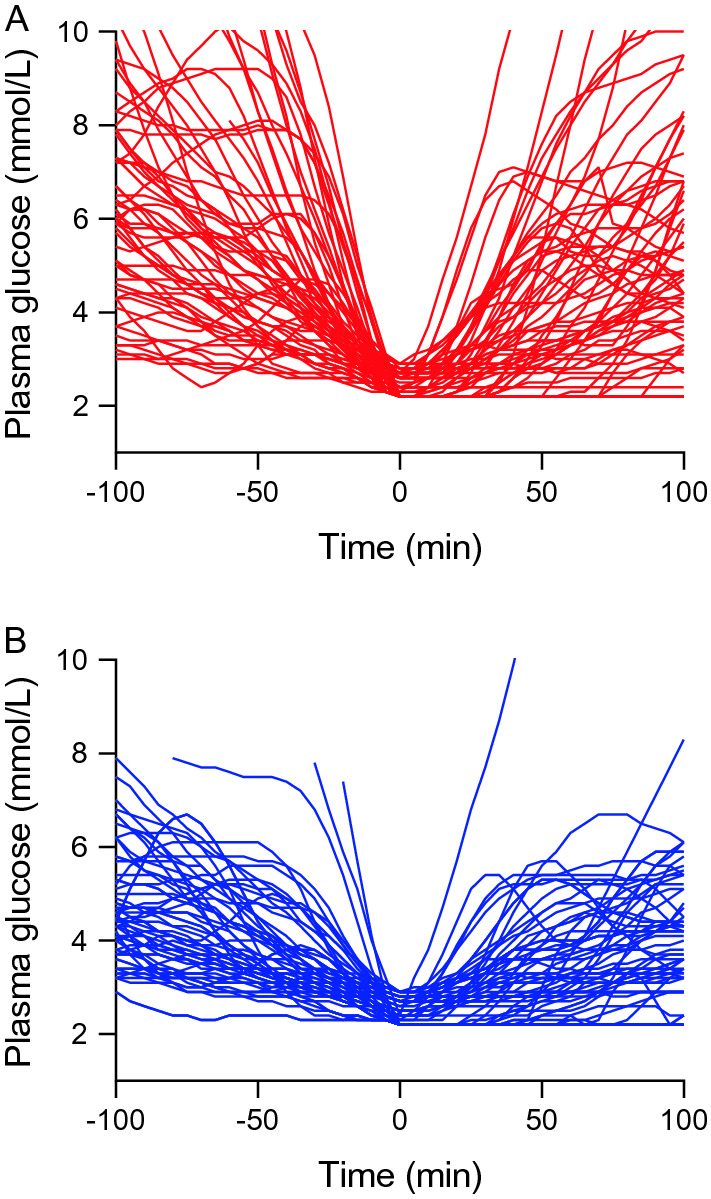
Glucose profiles during episodes of level 2 hypoglycemia. **A** Daytime and **B** nighttime plasma glucose profiles during episodes of level 2 hypoglycemia (plasma glucose nadir < 3.0 mmol/L). Time 0 is the time of plasma glucose nadir defined as first measurement equal to the minimum measured plasma glucose value during the hypoglycemic episode

In total, 2470 days of valid glucose recordings were obtained with a mean of 118 ± 6 days per patient. Time spent in hypoglycemia (level 1 + level 2), target range and hyperglycemia (level 1 + level 2) were 0.5% (0.3–1.1), 74.3% (68.0–79.3) and 24.8% (17.2–29.9), respectively (Table 2, Fig. 1). One patient did not have any measurements in the hypoglycemic range. Time in hypoglycemia was markedly greater in patients treated with basal/bolus insulin regimen or mixed insulin compared to basal insulin only (1.8% [0.5–4.2] and 0.3% [0.1–0.8], respectively), and time in hypoglycemia was higher during nighttime when compared to daytime (0.7% [0.7–2.7] and 0.4% [0.2–0.8], respectively). Nighttime episodes had a longer duration than daytime episodes, whereas plasma glucose nadir was slightly lower during daytime hypoglycemia (Table 3, Fig. 2). Glycemic variability as estimated by CV was 28.8% (25.9–35.1) and 16 out of 21 patients (76.2%) had a CV less than 36%.

**Table 2:** Glycemic metrics obtained by continuous glucose monitoring

	Daytime	Nighttime	24 h
Mean glucose (mmol/L)	9.0 [8.5; 9.4]	7.5 [7.1; 7.9]	8.6 [8.2; 9.0]
Glycemic variability			
Standard deviation	2.6 [2.3; 2.9]	2.1 [1.9; 2.3]	2.6 [2.3; 2.9]
Coefficient of variation (%)	27.9 (25.0–35.2)	26.3 (23.0–33.7)	28.8 (25.8–35.1)
Time in ranges (%)			
Time in level 2 hypoglycemia (< 3.0 mmol/L)	0.1 (0.0–0.3)	0.2 (0.0–0.7)	0.1 (0.0–0.3)
Time in level 1 hypoglycemia (3.0–3.8 mmol/L)	0.3 (0.1–0.7)	0.7 (0.4–2.0)	0.5 (0.2–0.9)
Time in range (3.9–10.0 mmol/L)	71.2 (62.0–77.9)	84.9 (77.6–88.1)	74.3 (68.0–79.3)
Time in level 1 hyperglycemia (10.1–13.9 mmol/L)	24.5 (17.1–27.2)	11.1 (6.4–15.2)	20.6 (14.2–22.4)
Time in level 2 hyperglycemia (> 13.9 mmol/L)	3.9 (1.4–6.1)	0.9 (0.4–1.4)	3.0 (1.3–4.8)

Continuous data are presented as median with interquartile range in parentheses or mean with 95% CIs

**Table 3:** Characterization of daytime and nighttime episodes of hypoglycemia

	Daytime	Nighttime
Episodes of hypoglycemia (N)	320	207
Level 1 (N)	208	149
Level 2 (N)	112	58
Patients with hypoglycemic episodes (N)	18	19
Episodes per patient	9 (6–32)	5 (2–20)
Duration (min)	55 [48; 64]	74 [64; 87]
Plasma glucose nadir (mmol/L)	3.1 [3.0; 3.2]	3.3 [3.2; 3.3]

Continuous data are presented as median with interquartile range in parentheses or mean with 95% CIs

### Cardiac arrhythmias

In total, 2415 episodes of potential cardiac arrhythmias were evaluated. Clinically significant arrhythmias as defined by protocol were experienced by 12 (57%) patients with the number of episodes per patient ranging from 1 to 522. Pauses and atrial fibrillation accounted for 554 (77%) and 162 (22%) of the episodes, respectively (Fig. 1). Furthermore, two episodes of bradycardia, five episodes of non-sustained ventricular tachycardia and one episode of sustained ventricular tachycardia were recorded. The incidence of cardiac arrhythmias was higher during nighttime than daytime (IRR 4.22 [3.48–5.15]). None of the reported episodes were symptomatic. Detection of subclinical atrial fibrillation by the ICMs resulted in therapeutic intervention with oral anti-coagulation therapy in four participants during the study period. The patient experiencing sustained ventricular tachycardia was referred to clinical evaluation including coronary angiography. None of the remaining episodes resulted in clinical intervention.

### Cardiac arrhythmias and hypoglycemia

**Fig. 3:**
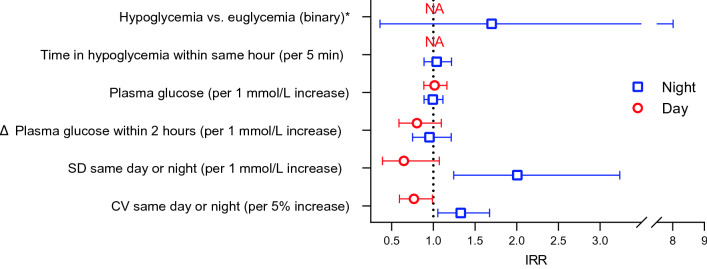
Hypoglycemia, glycemic variability and risk of cardiac arrhythmias. Incident rate ratio (95% CI) for cardiac arrhythmia (as defined by the study protocol) according to within-subject change in glycemic summaries (generalized linear mixed model). Note that no arrhythmias were detected during daytime hypoglycemia. *Risk of arrhythmia during an hour with occurrence of hypoglycemia defined as plasma glucose < 3.9 mmol/L compared with an hour of euglycemia with plasma glucose 3.9–10.0 mmol/L. Abbreviations: CV, coefficient of variation; NA, not applicable; SD, standard deviation

CGM data were recorded for 207 (29%) episodes of cardiac arrhythmia within 141 different individual observation hours. There was no incidence of simultaneous hypoglycemia and cardiac arrhythmia during daytime. During nighttime, there was no difference in the incidence rate of cardiac arrhythmias during hypoglycemia when compared to euglycemia (IRR 1.70 [0.36–8.01]) (Fig. 3). No effect of time in hypoglycemia within same hour on the incidence rate of cardiac arrhythmias was observed (IRR 1.04 [0.89–1.22] per 5 min). No difference in the incidence rate of cardiac arrhythmias with changes in plasma glucose compared to the subject-specific mean was found during daytime (IRR 1.02 [0.89–1.16] per 1 mmol/L increase) or nighttime (IRR 0.99 [0.89–1.11] per 1 mmol/L increase).

### Cardiac arrhythmias and glycemic variability

When applying CV as a measure of glycemic variability, which takes into account the skewed distribution of plasma glucose data, incidence rate of cardiac arrhythmias decreased with increasing CV during daytime, whereas the incidence rate of cardiac arrhythmias increased with increasing CV during nighttime (IRR 0.77 [0.59–0.99] and IRR 1.33 [1.05–1.67] per 5% absolute increase, respectively). Similarly, a sensitivity analysis applying SD showed a tendency to an inverse relation between SD and incidence rate of cardiac arrhythmias during daytime (IRR 0.65 [0.39–1.07]), whereas the incidence rate of cardiac arrhythmias increased with increasing SD during nighttime (IRR 2.01 [1.24–3.24] per 1 mmol/L). The two-hour absolute excursion in plasma glucose had no significant effect on the incidence rate of cardiac arrhythmias during daytime (IRR 0.80 [0.59–1.10] per 1 mmol/L increase) or nighttime (IRR 0.95 [0.75–1.21] per 1 mmol/L increase) (Fig. 3).

### Cardiac arrhythmias and hyperglycemia

**Fig. 4:**
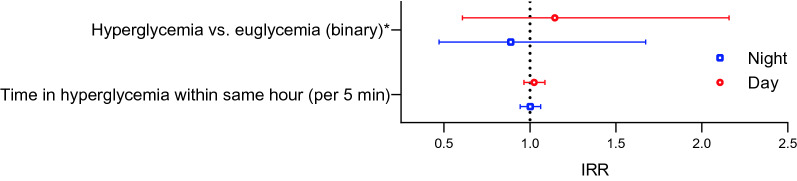
Hyperglycemia and risk of cardiac arrhythmias. Incident rate ratio (95% CI) for cardiac arrhythmia (as defined by the study protocol) according to within-subject change in glycemic summaries of hyperglycemia (generalized linear mixed model). *Risk of arrhythmia during an hour with occurrence of hyperglycemia defined as plasma glucose > 10.0 mmol/L compared with an hour of euglycemia with plasma glucose 3.9–10.0 mmol/L

There was no difference in the incidence rate of cardiac arrhythmias during hyperglycemia when compared to euglycemia during daytime (IRR 1.14 [0.61–2.16]) or nighttime (IRR 0.89 [0.47–1.67]) (Fig. 4). Similarly, time in hyperglycemia within same hour was not associated with incidence of cardiac arrhythmias (IRR 1.02 [0.96–1.09] for daytime and 1.00 [0.94–1.06] for nighttime).

## Discussion

The present study aimed to investigate the association between hypoglycemia, glycemic variability and cardiac arrhythmias in patients with type 2 diabetes by applying long-term cardiac and glycemic monitoring. To our knowledge, the study provides the most extensive data set on concomitant monitoring of glycemia and cardiac rhythm to date. Although clinically relevant arrhythmias were common in this population of insulin-treated patients with type 2 diabetes without any pre-existing symptoms or diagnoses of arrhythmia, no association between hypoglycemia and cardiac arrhythmias was found. However, data revealed an association between glycemic variability and cardiac arrhythmias with a difference between daytime and nighttime. During nighttime, incidence rate of cardiac arrhythmias increased with increasing glycemic variability within same nighttime period, whereas it decreased during daytime. Our findings suggest that that glycemic variability serves an independent predictor of cardiac arrhythmias and not merely a predictor of hypoglycemia.

### Glycemic variability and cardiac arrhythmias—clinical implications

Multiple studies have demonstrated an association between long-term glycemic variability expressed as variability in HbA1c/fasting plasma glucose and risk of cardiovascular disease including myocardial infarction, stroke, all-cause mortality and left ventricular adverse remodeling after STEMI [22–25]. Furthermore, short-term glycemic variability assessed by CGM has been associated with increased coronary plaque vulnerability, high platelet reactivity and increased risk of major adverse cardiovascular events in patients with myocardial infarction [26–30]. The present study adds to current evidence linking glycemic variability to cardiovascular disease by demonstrating a temporal relation between short-term glycemic variability and cardiac arrhythmias. The association between glycemic variability and cardiac arrhythmias is unlikely to be explained by increased incidence of hyperglycemia since no association between hyperglycemia and incidence of cardiac arrhythmias was detected. The temporal relation indicates that glycemic variability may not merely be a predictor of cardiovascular disease. Nevertheless, long-term randomized intervention studies are necessary to determine if reducing glycemic variability may ameliorate cardiovascular complications in patients with diabetes. These studies are difficult to perform since pharmacological modulation of glycemic variability without affecting other important glycemic metrics (e.g., hyperglycemia) is difficult [22]. In clinical practice, glycemic variability is increasingly recognized as an important glycemic metric and international consensus on reporting and interpretation has been established [18]. In patients with type 2 diabetes, glycemic variability may be reduced by the use of glucose-dependent glucose-lowering drugs (e.g., metformin, dipeptidyl-peptidase 4 inhibitors (DPP-4is), sodium-glucose co-transporter 2 inhibitors (SGLT2is) and glucagon-like peptide 1 receptor agonists (GLP-1RAs)) and lifestyle changes (e.g., low-carbohydrate diet and exercise) [31].

### Glycemic variability and cardiac arrhythmias—mechanistic insight

A temporal association between glycemic variability and cardiac arrhythmias has not previously been reported. However, diurnal differences in the association between glycemia and rhythm abnormalities are supported by previous studies. These studies indicate that prolonged episodes of nocturnal hypoglycemia may result in sympathetic withdrawal and over-compensatory vagal counteraction, which may result in bradycardia and, potentially, brady-arrhythmias [8, 11]. In the present study, the risk of arrhythmias was higher during nighttime with most events being pauses or atrial fibrillation. This confirms previous findings of an increased incidence of brady-arrhythmias and atrial fibrillation at night [32, 33]. This circadian rhythm is thought to be controlled from a central clock in the suprachiasmatic nucleus, which modulates conduction and expression of cardiac ion channels through the autonomic nervous system with increased nocturnal vagal activity [34]. Although no association between hypoglycemia and cardiac arrhythmias was found in the present study, an increased vagal tone during prolonged nocturnal episodes of hypoglycemia is likely to enhance the circadian rhythm of cardiac arrhythmias and predispose to nocturnal episodes of bradyarrhythmia and atrial fibrillation [8, 34, 35]. Similarly, an effect of glycemic variability on risk of cardiac arrhythmias could potentially be mediated through modulation of the autonomic nervous system, which could also explain the observed differences between daytime and nighttime. Whereas increased glycemic variability is associated with the presence of cardiac autonomic neuropathy [36], the acute effect of increased glycemic variability on vagal tone has not been investigated. Hence, it is unclear whether a modulation of the autonomic nervous system could explain the apparent effect of short-term glycemic variability on incidence of arrhythmias. Interestingly, increased long-term glycemic variability has also been associated with increased left ventricular mass and decreased left ventricular ejection fraction, which may increase the risk of cardiac arrhythmias [37].

### Hyperglycemia and cardiac arrhythmias

Daytime hyperglycemia has previously been reported to be associated with a lower incidence of atrial ectopic beats and complex ventricular premature beats but with a higher incidence of ventricular premature beats [8]. In the present study, no association between hyperglycemia and cardiac arrhythmias was found. This finding supports that glycemic variability does not merely serve as a marker of hyperglycemia, but that the association between glycemic variability and cardiac arrhythmias is independent of absolute plasma glucose levels.

### Hypoglycemia and cardiac arrhythmias

In the present study, we chose to include insulin-treated patients with an HbA1c ≤ 7.5% to ensure a population with high risk of hypoglycemia. However, time in hypoglycemia was relatively low compared with previous studies [8, 38, 39]. Notably, patients treated with long-acting basal insulins had relatively little time in hypoglycemia and, consequently, the hypoglycemic exposure was less than expected. With the development of new classes of glucose-lowering drugs for the treatment of type 2 diabetes with a glucose-dependent mode of action and ultra-long-acting basal insulins, risk of hypoglycemia in patients with type 2 diabetes is likely to have been reduced compared to previous trials investigating the effect of near-normal HbA1c targets in type 2 diabetes [40–44]. In previous studies, cardiovascular disease has been linked to episodes of severe (level 3) hypoglycemia [45, 46], whereas episodes of hypoglycemia in the present study were none-severe (level 1 or level 2). Accordingly, episodes of hypoglycemia recorded in the present study may not have been severe enough to provoke cardiac arrhythmias. As the incidence of severe hypoglycemia is higher in patients with type 1 diabetes [47], future studies applying long-term glycemic and cardiac monitoring in these patients are warranted.

### Limitations

Due to the limited sensor life of the applied CGM system and the study burden to the participants, CGM data were only obtained for one third of the observation period. Furthermore, recruitment was difficult since the study was demanding for the participants and as insulin treatment in our outpatient clinic is becoming increasingly rare due to new treatment options. This resulted in a relatively small sample size and correspondingly wide confidence intervals for the effects of interest. Accordingly, the present study does not exclude an association between hypoglycemia and cardiac arrhythmias or determine if glycemic variability is better predictor of cardiac arrhythmias. Four participants received beta blockers and four participants received SGLT2is, which both may have decreased the incidence of cardiac arrhythmias [48–51]. However, as participants served as their own controls, this has not affected the association between glycemic predictors and the outcome of arrhythmia.

## Conclusions

Asymptomatic cardiac arrhythmias are common in insulin-treated patients with longstanding type 2 diabetes and good glycemic control. We did not find an association between hypoglycemia and episodes of cardiac arrhythmia, whereas glycemic variability was associated with the incidence of arrhythmias with distinctive diurnal differences. However further studies are needed to elucidate the relation between glycemic variability and cardiac arrhythmias. Although challenging from a feasibility perspective, future studies investigating the relationship between hypoglycemia, glycemic variability and cardiac arrhythmias should focus on large-scale, long-term concomitant glycemic and cardiac monitoring in patients with type 2 diabetes as well as in patients with type 1 diabetes.

## Supplementary Information

**Additional file 1. **Reveal LINQ setup.

## Data Availability

The datasets generated and analyzed during the current study are not publicly available due Danish data protections laws but are available from the corresponding author on reasonable request.

## Abbreviations

CV: Coefficient of variation
DPP-4is: Dipeptidyl-peptidase 4 inhibitors
GLP-1RAs: Glucagon-like peptide 1 receptor agonists
ICM: Implantable cardiac monitors
IRR: Incidence rate ratio
SD: Standard deviation
SGLT2is: Sodium-glucose co-transporter 2 inhibitors
