# Supplementary material for: Associations of hypoglycemia, glycemic variability and risk of cardiac arrhythmias in insulin-treated patients with type 2 diabetes: a prospective, observational study
Source: Cardiovasc Diabetol. 2021 Dec 24;20:241. doi: 10.1186/s12933-021-01425-0 (PMC8710000; doi:10.1186/s12933-021-01425-0)
Supplement: Supplementary file 1 — Additional file 1. Reveal LINQ setup. [file 12933_2021_1425_MOESM1_ESM.docx]

**Additional file: Table S1** Reveal LINQ setup.

| Reasons for monitoring | Ventricular tachycardia |
| --- | --- |
| Sensitivity | 0.035 mV |
| Blanking | 150 ms |
| Sensing threshold decay delay | 150 ms |
| Tachy | On, 0.85% of max heart rate |
| FVT zone (non-programmable) | On, 260 ms (231 bpm), 30/40 beats |
| Brady | On, 2000 ms (30 bpm), 8 beats |
| Pause | On, 3 sec. |
| AT/AF | AF only |
| Wireless data | Tachy, brady, pause |
| *AT/AF detection* |  |
| AT/AF detection | On |
| Type | AF only |
| AF detection | Least sensitive |
| Ectopy rejection | Aggressive |
| AT/AF recording threshold | ≥ 6 min. |

*An individualized detection heart rate determined as 85% of maximal heart rate calculated by the formula 208-0.7×age (1). The tachycardia detection rate could be increased in two steps to 95% og maximal heart rate and 230-age, respectively, if the number of false positive reports was ≥1 per week during the three-week run-in period.

**Reference**

1. Tanaka H, Monahan KD, Seals DR. Age-predicted maximal heart rate revisited. J Am Coll Cardiol. 2001 Jan;37(1):153–6.
